# Supplementary material for: Exploring the virulence gene interactome with CRISPR/dCas9 in the human malaria parasite
Source: Mol Syst Biol. 2020 Aug 20;16(8):e9569. doi: 10.15252/msb.20209569 (PMC7440042; doi:10.15252/msb.20209569)
Supplement: Supplementary file 1 — Expanded View Figures PDF [file MSB-16-e9569-s001.pdf]

# Expanded View Figures

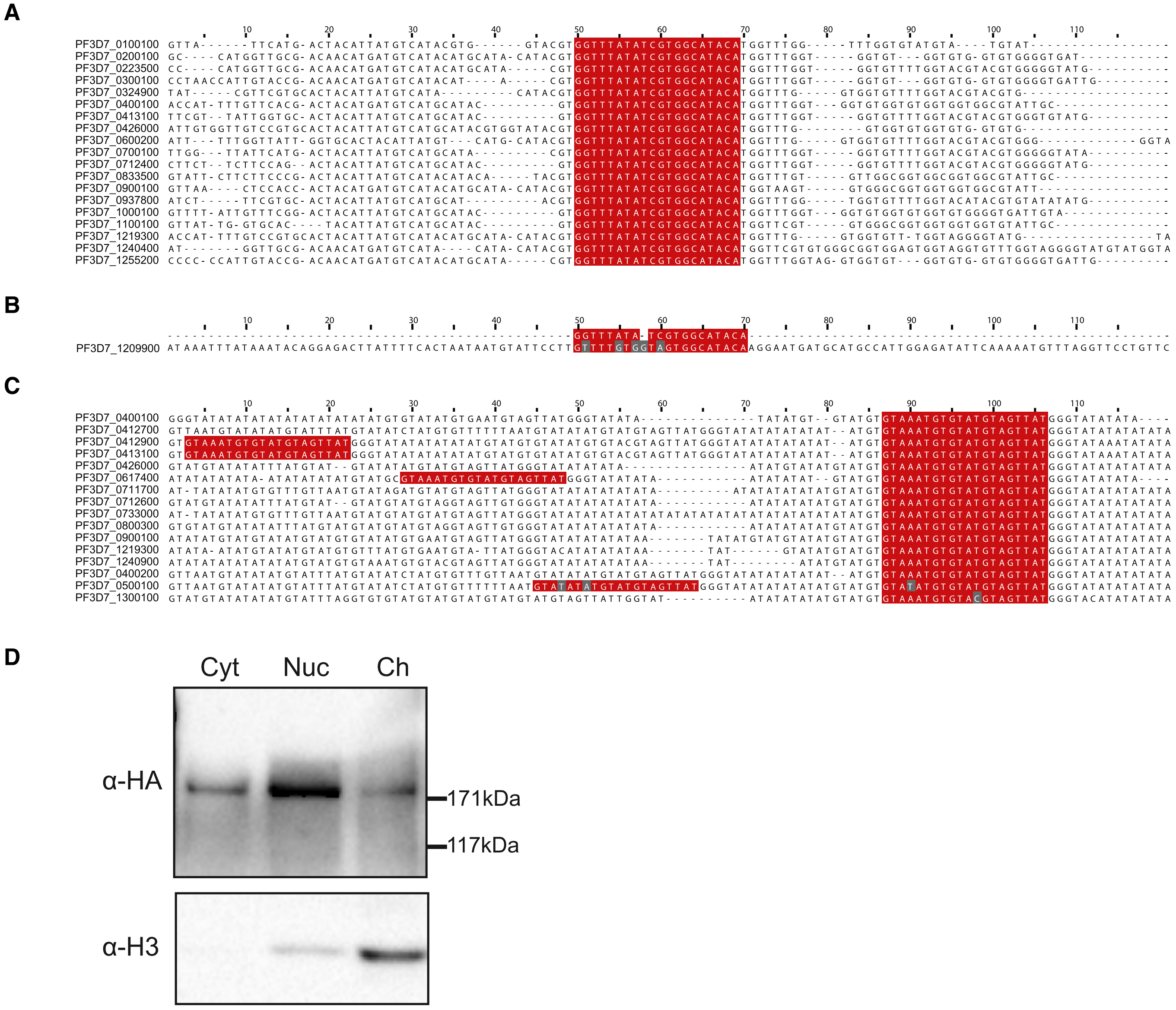


**Figure EV1. Supplementary information for immunoprecipitation of non-targeted and *var* gene-targeted dCas9.**

A–C Alignments of the *var* gene promoter (A) and intron (C) non-coding strand sequences, with sgRNA sequences highlighted in red. The presumed off-target sequence for the *var* promoter-targeted dCas9 is shown in (B). Mismatches are highlighted in gray. Corresponding data for the promoter or intron sgRNAs can be found in Tables EV1 and EV2, respectively.

D Western blot analysis of cell fractions from ring stage parasites of the non-targeted dCas9 control strain. Levels of dCas9-3HA and histone H3 in the cytoplasmic (Cyt), nuclear (excluding chromatin, Nuc), and chromatin (Ch) fractions are revealed with anti-HA and anti-H3 antibodies, respectively. Molecular weights are shown to the right.

Source data are available online for this figure.

© 2020 The Authors 

**Figure EV2. *var* gene transcription in non-targeted and *var* gene-targeted dCas9 strains.**

A Circos plot of ChIP-seq data showing genome-wide enrichment of non-targeted dCas9 in ring stage parasites. The 14 chromosomes are represented circularly by the outer gray bars, with chromosome number indicated in roman numerals and chromosome distances indicated in Arabic numerals (Mbp). Enrichment (ChIP normalized to input) is shown as average reads per million (RPM) over bins of 1,000 nt. The maximum *y*-axis value is 1,000 RPM (rings represent increments of 167). *var* genes are represented by red bars. One replicate was used for the dCas9 ChIP-seq.

B RNA-seq data from clonal parasite populations of promoter- (red) or intron-targeted (blue) dCas9 strains show transcript abundance (*y*-axis = RPKM) for all *var* genes (*x*-axis) at 14 hpi. One replicate was performed for each clone.

C Cell cycle progression estimation of synchronized non-clonal bulk parasite cultures of the promoter- (red), intron- (blue), and non-targeted (gray) dCas9 strains at 14 hpi. RNA-seq data from these parasites were compared to microarray data from Bozdech *et al* (2003) (Data ref: Bozdech *et al*, 2003) as in Lemieux *et al* (2009).

D RNA-seq data from non-clonal bulk parasite cultures of the promoter- (red), intron- (blue), and non-targeted (gray) dCas9 strains at 14 hpi show transcript abundance (*y*-axis = RPKM) for all *var* genes (*x*-axis). The gray dotted boxes indicate *var* genes bound by dCas9 in the promoter-targeted strain (top graph) or intron-targeted strain (bottom graph). One replicate was performed for each strain.

E Circos plot of dCas9 ChIP-seq (outer ring in red) and RIP-seq (inner ring in green) data showing genome-wide DNA and RNA enrichment, respectively, in *var* gene promoter-targeted dCas9 immunoprecipitation at 14 hpi. The 14 chromosomes are represented circularly by the outer gray bars, with chromosome number indicated in roman numerals and chromosome distances indicated in Arabic numerals (Mbp). dCas9 ChIP enrichment (input-subtracted and normalized to the corresponding value for the non-targeted dCas9 control) is shown as average RPM over bins of 1,000 nt. dCas9 RIP enrichment (IgG-subtracted and normalized to the corresponding value for the non-targeted dCas9 control) is shown as average enrichment per gene. The maximum *y*-axis value is 3,000 for ChIP-seq, and 3 for RIP-seq. *var* genes are represented by red bars. One replicate was performed for ChIP-seq, and one replicate was performed for HA and IgG control and promoter-targeted dCas9 RIP-seq. ChIP-seq peak quantification can be found in Table EV1, and RIP-seq quantification can be found in Table EV3.

►


© 2020 The Authors

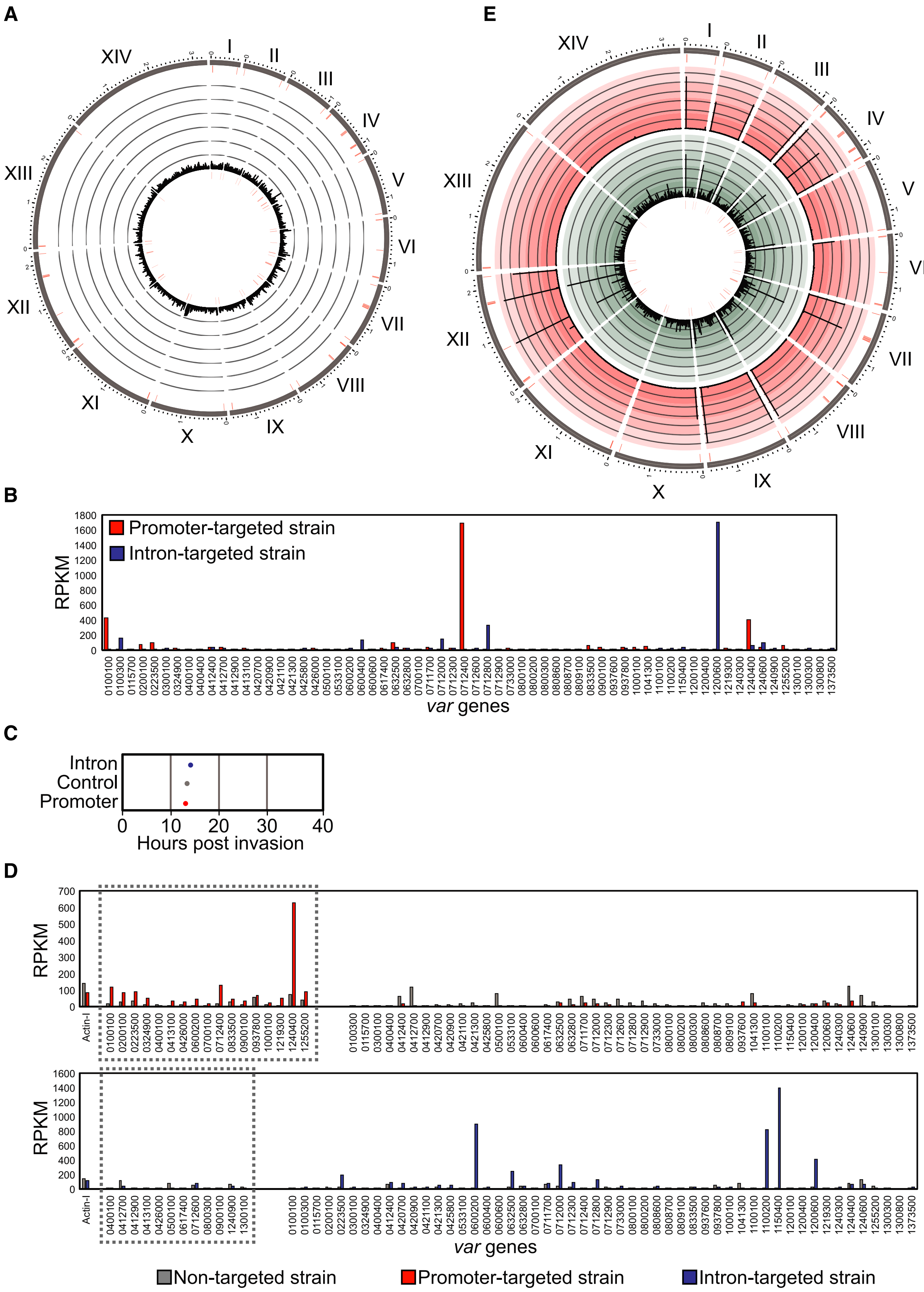
A
XIV
I
II
III
IV
V
VI
VII
VIII
IX
X
XI
XII
XIII
E
B
Promoter-targeted strain
Intron-targeted strain
RPKM
var genes
C
Intron
Control
Promoter
0
10
20
30
40
Hours post invasion
D
RPKM
Actin-I
var genes
Non-targeted strain
Promoter-targeted strain
Intron-targeted strain

**Figure EV2.**

**Figure EV3. Supplementary information for proteomic analysis of non-targeted and *var* gene-targeted dCas9 immunoprecipitation.**

A Graph showing dCas9 peptide ions (*x*-axis) versus total proteins (*y*-axis) detected in the proteomic analysis for each of four replicates of the intron- (blue circles), promoter- (red triangles), or non-targeted (gray squares) dCas9 immunoprecipitations. Corresponding data can be found in Table EV4 (top).

B Agilent DNA high sensitivity 2100 Bioanalyzer trace showing a representative distribution of DNA fragments after sonication of the chromatin samples used for dCas9 immunoprecipitation. *x*-axis indicates DNA fragment size (bp), and *y*-axis indicates abundance (fluorescence units). Lower DNA marker is shown in green, and upper DNA marker is shown in purple. A representative gel is shown at the bottom.

C ChIP-seq data showing enrichment of dCas9 in the *var* gene promoter-targeted strain. Genome location is indicated at the top. The *x*-axis is DNA sequence, with a representative subtelomeric *var* gene (PF3D7_1000100) represented by black boxes indented to delineate introns and labeled with white arrowheads to indicate transcription direction. The *y*-axis is input-subtracted ChIP enrichment (peak $q = 5.54 \times 10^{-275}$). SPE2 DNA sequences identified in Flueck *et al* (2010) are indicated with gray lines. One replicate was performed for ChIP-seq.

D Phylogenetic tree comparing *Pf*ISWI with SNF2 domain-containing chromatin remodelers in *P. falciparum* and other organisms (indicated in italicized letters) based on the SNF2 domain sequence (left). Bootstrap values of 1,000 replicates are shown at nodes. General representations of protein compositions are shown on the right, with SNF2 and helicase (“Hel”) domains in gray. The ISWI family of chromatin remodelers contains HAND, SANT, and SLIDE domains (green), the SWI/SNF family contains a bromodomain (blue), and the CHD family contains chromodomains (orange). *Pf*ISWI contains zinc finger domains (red).

E Graphical representation of MORC family proteins in *P. falciparum* and other eukaryotes. S5 = S5 fold domain, ZF = zinc finger domain. Black box = coiled-coil domain.

F Odds ratios and *P*-values calculated with Fisher’s exact test (with GeneOverlap R package) for protein overlap (Venn diagram in Fig 3C) between the ISWI IP LC-MS/MS, *var* promoter-targeted dCas9 LC-MS/MS (compared to intron-targeted dCas9), or *var* promoter-targeted dCas9 LC-MS/MS (compared to control dCas9). Odds ratio represents strength of association ($\leq 1$ indicates no association between two lists while $> 1$ indicates association).

 © 2020 The Authors

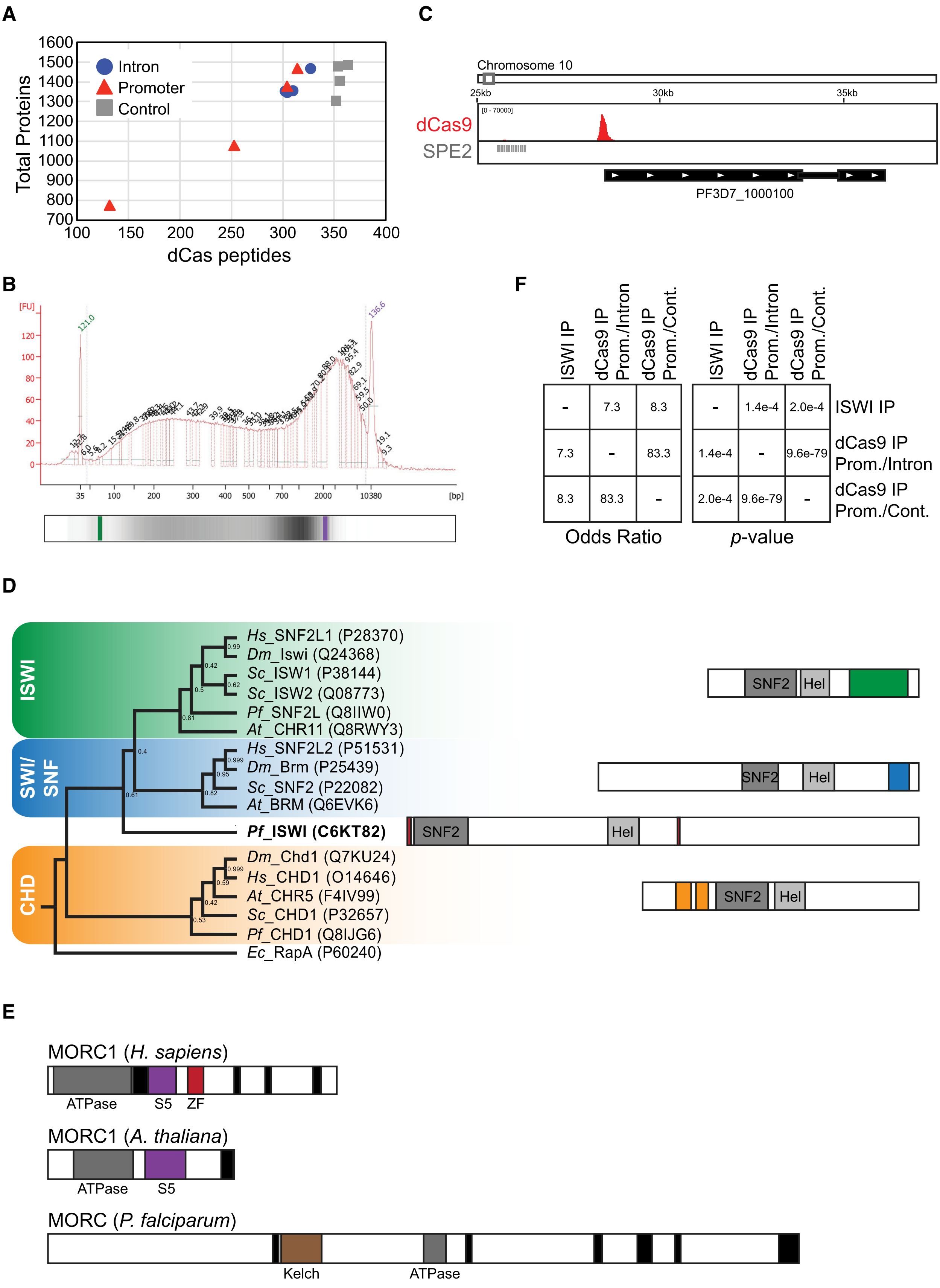
A
Intron
Promoter
Control
Total Proteins
dCas peptides
1600
1500
1400
1300
1200
1100
1000
900
800
700
100
150
200
250
300
350
400
C
Chromosome 10
25kb
30kb
35kb
dCas9
SPE2
[0 - 70000]
PF3D7_1000100
B
[FU]
121.0
136.6
[bp]
35
100
200
300
400
500
700
2000
10380
F
ISWI IP
dCas9 IP Prom./Intron
dCas9 IP Prom./Cont.
- 7.3 8.3
7.3 - 83.3
8.3 83.3 -
- 1.4e-4 2.0e-4
1.4e-4 - 9.6e-79
2.0e-4 9.6e-79 -
Odds Ratio
p-value
D
ISWI
SWI/ SNF
CHD
Hs_SNF2L1 (P28370)
Dm_Iswi (Q24368)
Sc_ISW1 (P38144)
Sc_ISW2 (Q08773)
Pf_SNF2L (Q8IIW0)
At_CHR11 (Q8RWY3)
Hs_SNF2L2 (P51531)
Dm_Brm (P25439)
Sc_SNF2 (P22082)
At_BRM (Q6EVK6)
Pf_ISWI (C6KT82)
Dm_Chd1 (Q7KU24)
Hs_CHD1 (O14646)
At_CHR5 (F4IV99)
Sc_CHD1 (P32657)
Pf_CHD1 (Q8IJG6)
Ec_RapA (P60240)
SNF2
Hel
E
MORC1 (H. sapiens)
ATPase
S5
ZF
MORC1 (A. thaliana)
ATPase
S5
MORC (P. falciparum)
Kelch
ATPase

**Figure EV3.**

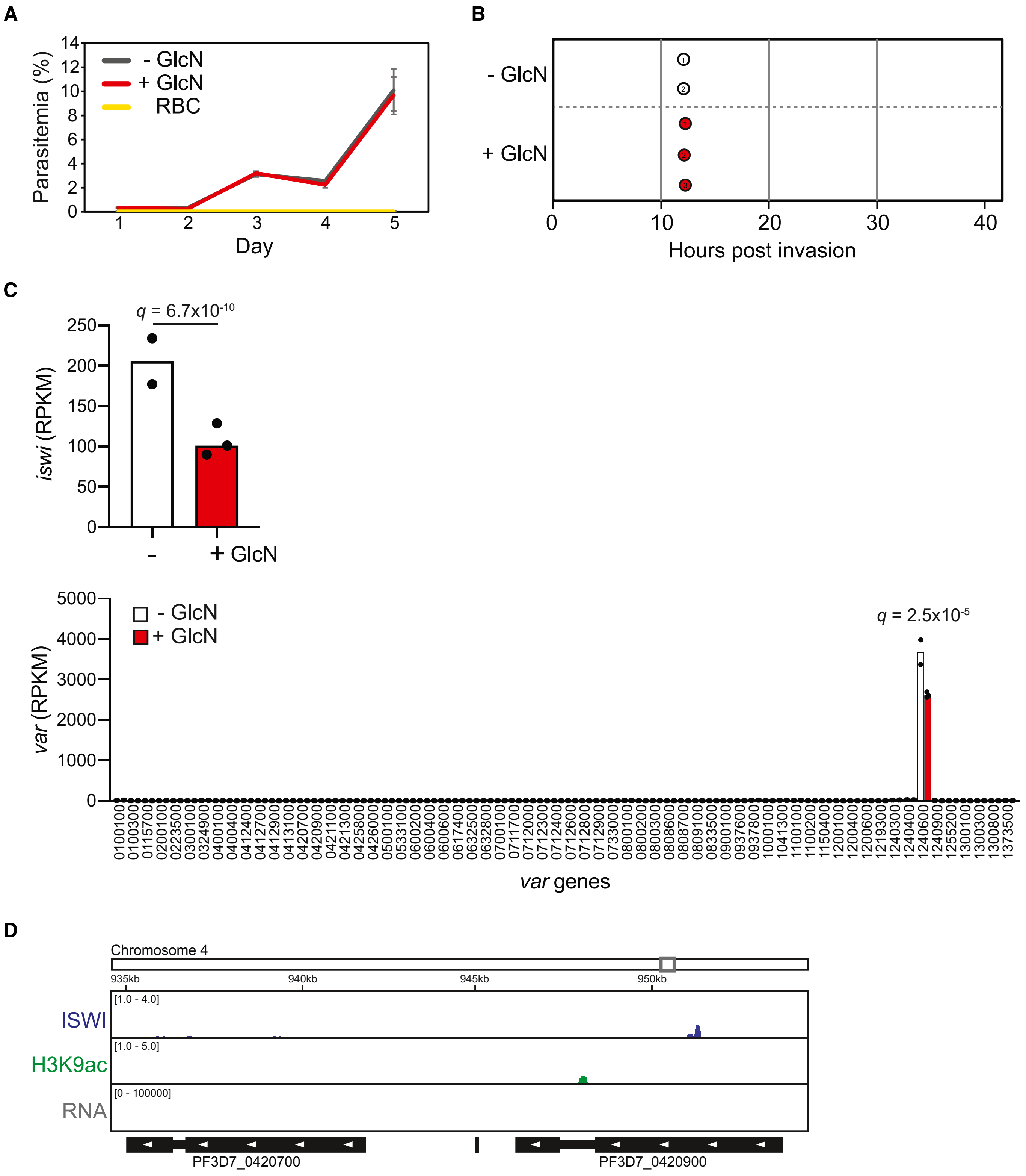


 © 2020 The Authors

**Figure EV4. Supplementary information for differential expression analysis of ISWI knockdown and ISWI ChIP sequencing.**

A Growth curve over 5 days of clonal ISWI-3HA-ribo parasites in the absence ("− GlcN" in gray) or presence ("+ GlcN" in red) of glucosamine. Uninfected red blood cells ("RBC" in yellow) serve as reference of background. Error bars indicate standard deviation of three technical replicates in blood from two different donors ($n$ = 6).

B Cell cycle progression estimation of an ISWI-3HA-ribo parasite clone in the absence ("− GlcN" in white) or presence ("+ GlcN" in red) of glucosamine. RNA-seq data from synchronized parasites harvested at 12 hpi were compared to microarray data from Bozdech *et al* (2003) (Data ref: Bozdech *et al*, 2003) as in Lemieux *et al* (2009). Replicates are represented with numbered circles.

C RNA-seq of an ISWI-3HA-ribo clone at 12 hpi shows *iswi* (top, $q = 6.71 \times 10^{-10}$) and *var* gene (bottom, active *var* gene $q = 2.53 \times 10^{-5}$) transcript levels in the absence ("−", white) or presence ("+", red) of glucosamine (GlcN). $y$-axis is transcript level (RPKM). Bars represent averages of two and three replicates (individual values indicated with black dots) for untreated and glucosamine-treated parasites, respectively. $P$-values are calculated with a Wald test for significance of coefficients in a negative binomial generalized linear model as implemented in DESeq2 (Love *et al*, 2014). $q$ = Bonferroni corrected $P$-value. Corresponding data can be found in Source Data for Table EV12.

D ChIP-seq data show enrichment of ISWI (blue) and H3K9ac (green) in clonal ISWI-3HA parasites at 12 hpi at two silent *var* genes (PF3D7_0420700 and PF3D7_0420900). RNA-seq data from this clone at 12 hpi show transcript levels for these genes (gray). Genome location is indicated at the top of the panel. The $x$-axis is DNA sequence, with genes represented by black boxes indented to delineate introns and labeled with white arrowheads to indicate transcription direction. The $y$-axis is ChIP/Input for ChIP data and RPKM for RNA-seq data. One replicate was used for each ChIP-seq, and the RNA-seq data are from a single replicate from the untreated ISWI-3HA clone used for the differential expression analysis.

© 2020 The Authors
