# Supplementary figures and images for: Exploring the virulence gene interactome with CRISPR/dCas9 in the human malaria parasite
Source: Mol Syst Biol. 2020 Aug 20;16(8):e9569. doi: 10.15252/msb.20209569 (PMC7440042; doi:10.15252/msb.20209569)

Figure EV1D

$\alpha$ -HA

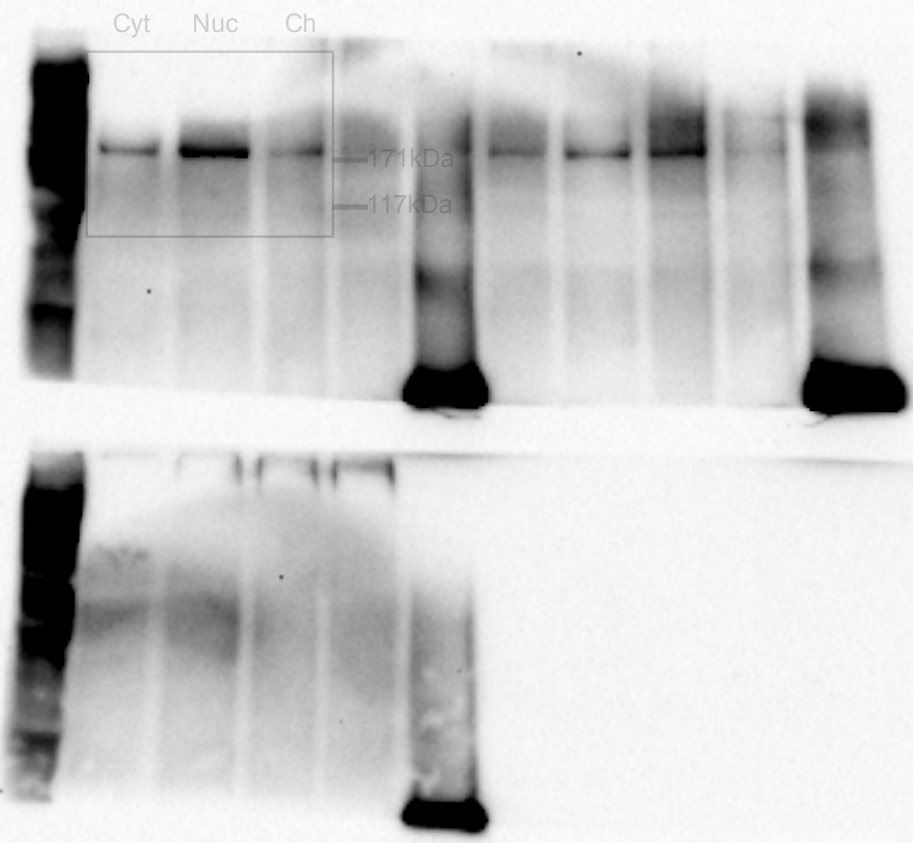

$\alpha$ -H3

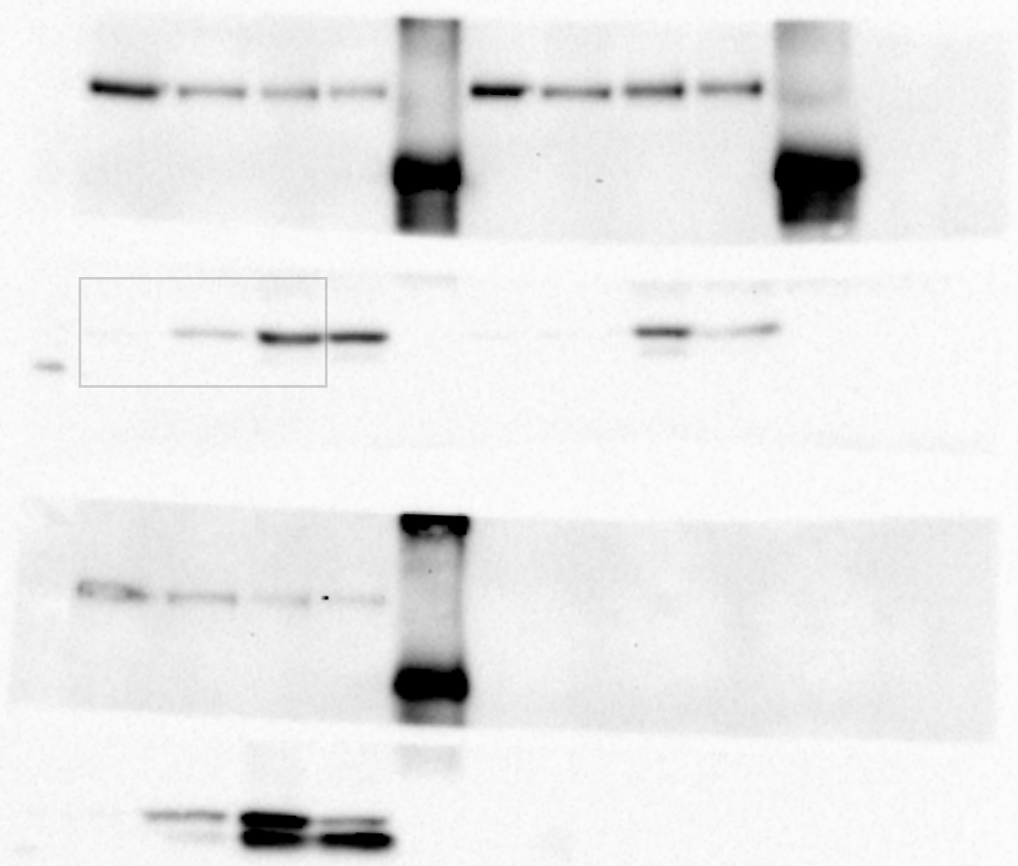

Supplement: Supplementary file 16 — Source Data for Expanded View [file MSB-16-e9569-s018.zip › Figure EV1D Source Data.pdf]

**Figure 1C**

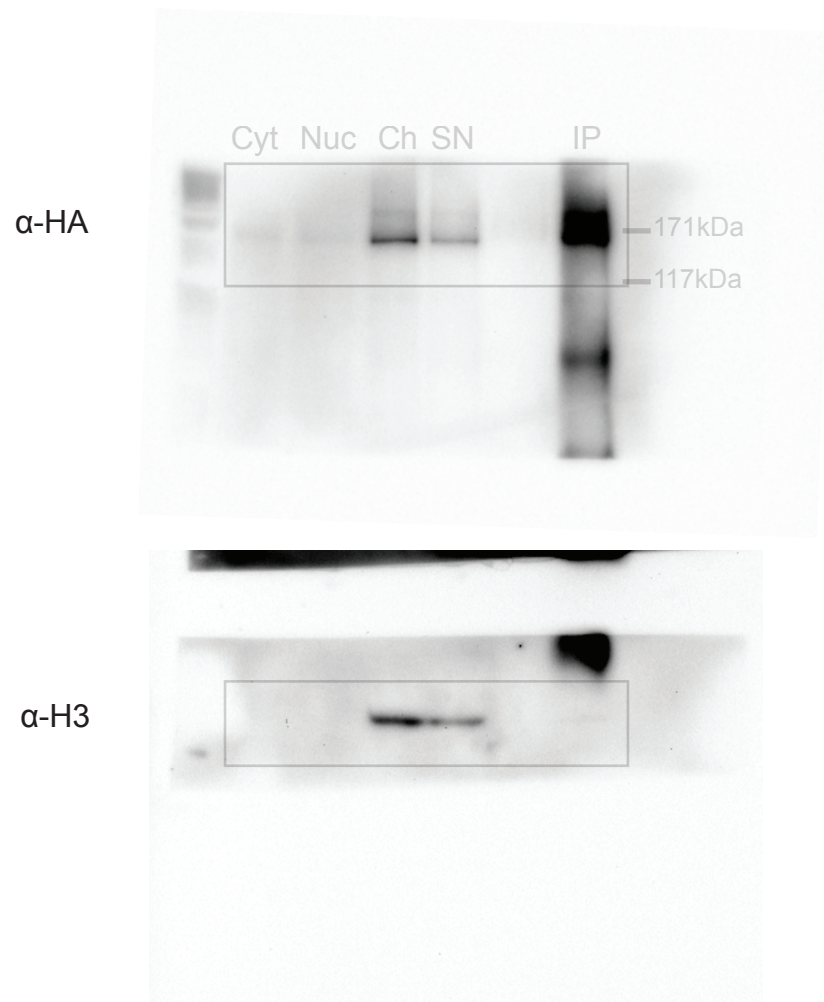

Supplement: Supplementary file 18 — Source Data for Figure 1 [file MSB-16-e9569-s016.pdf]

Figure 4A

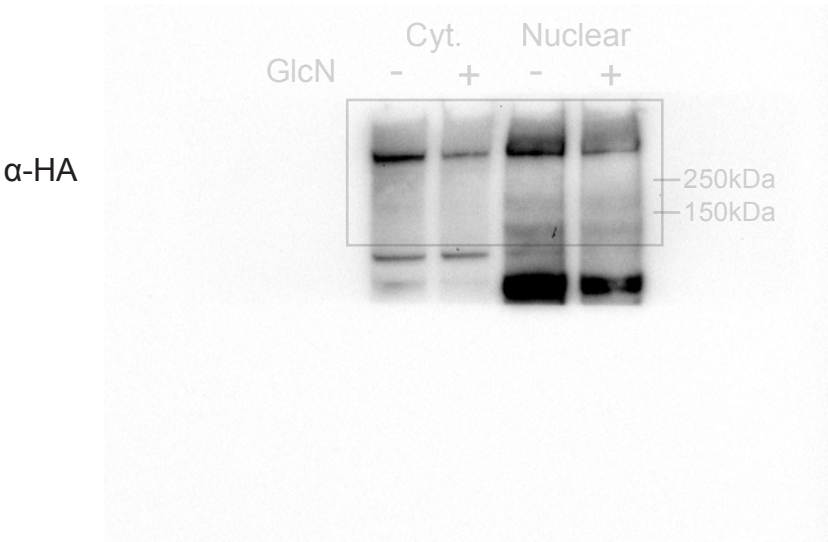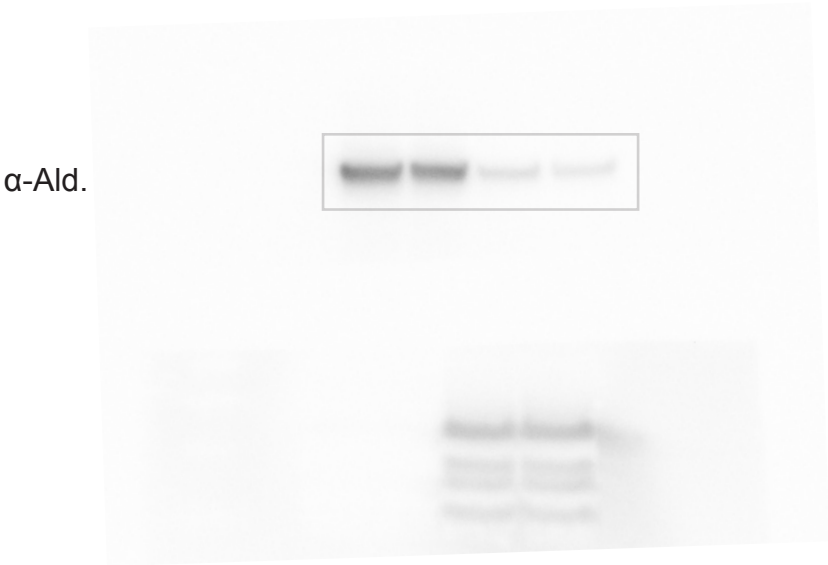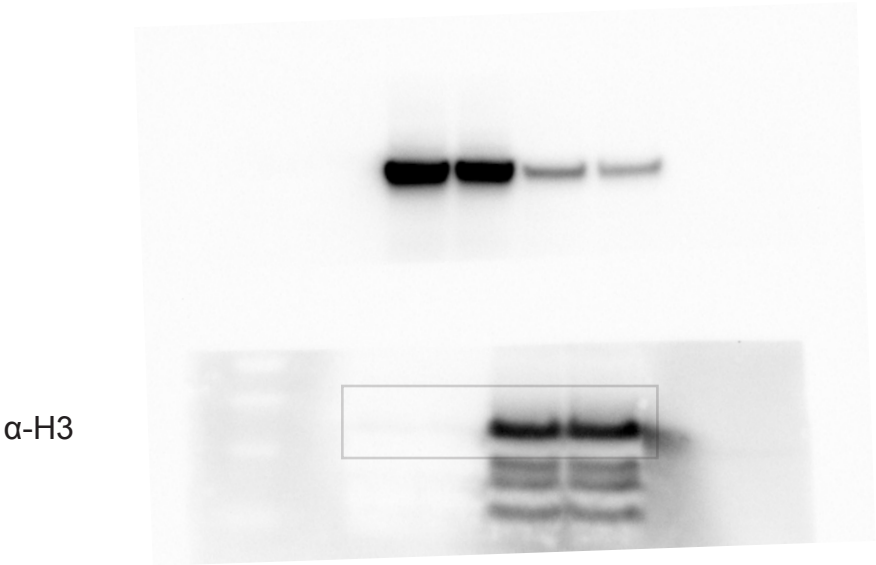

Supplement: Supplementary file 19 — Source Data for Figure 4 [file MSB-16-e9569-s017.pdf]
